# Weight dissatisfaction is linked to higher eating disinhibition and greater weight fluctuations in adults with weight management experience

**DOI:** 10.1177/13591053251338340

**Published:** 2025-05-29

**Authors:** Faranak Halali, Anja Lapveteläinen, Liisa I Tammela, Sari Hantunen, Raimo Lappalainen, Teuvo Kantanen, Leila Karhunen

**Affiliations:** 1 Institute of Public Health and Clinical Nutrition, Faculty of Health Sciences, University of Eastern Finland, Finland; 2 Department of Psychology, Faculty of Education and Psychology, University of Jyväskyä, Finland; 3 Department of Business, Faculty of Social Sciences and Business Studies, University of Eastern Finland, Finland

**Keywords:** disinhibition, psychological distress, rigid restraint, weight fluctuation, weight satisfaction

## Abstract

Body weight dissatisfaction has been associated with unfavorable eating behaviors such as eating disinhibition, more dieting, and poorer mental health. However, eating behavior and dietary correlates of weight satisfaction in adults is relatively understudied. This cross-sectional study investigated the associations of weight satisfaction with eating behavior dimensions (Three-Factor Eating Questionnaire-65), psychological distress (General Health Questionnaire-12), self-reported weight loss history, recent weight changes, and dietary intake. Regression analysis was used to identify predictors of weight dissatisfaction. Participants were 83 adults in Finland with experience in weight management. About two-thirds (55/83, 66%) were dissatisfied with their weight. The weight-dissatisfied group reported more disinhibition and rigid restraint of eating, higher body mass index, greater number of prior weight loss attempts, and more recent weight fluctuations. Eating disinhibition predicted weight dissatisfaction. The findings highlight the interrelations between weight dissatisfaction, disordered eating, and body weight as potential targets for weight management and health promotion.

## Introduction

Dissatisfaction with body weight is prevalent among adults, partly driven by gradual physical changes that occur in middle and later adulthood, such as weight gain and redistribution of fat to central areas of the body (Ponti et al., 2020). In a nationally representative study of Australian adults, 30% of men and 44% of women were dissatisfied with their weight (Feng and Wilson, 2019). A recent study among Brazilian adults reported that about 87% of women and 74% of men were dissatisfied with their body due to perceived excess weight, whereas over one-third of dissatisfied adults in both sexes did not actually present excess adiposity (Cabral et al., 2024). Among middle-aged and older women of the Swiss National Nutrition survey, 41% reported dissatisfaction with their weight (Chatelan and Carrard, 2021). Some studies report a stronger association between self-perceived weight status, rather than the actual weight status, and self-rated health and life satisfaction, highlighting that satisfaction with body weight engages a subjective element of body image (Duarte et al., 2021; Herman et al., 2013). Similarly, it has been reported that a more positive body image is associated with better wellbeing and quality of life (Goldschmidt et al., 2016; Griffiths et al., 2016).

Dissatisfaction with weight has been associated with greater body mass index (BMI; Silva et al., 2019), trying to alter weight or shape (Fredrickson et al., 2015), poorer quality of life (Olson, 2017), more sedentary lifestyle, higher dieting frequency (Blake et al., 2013), more prior attempts to lose weight (Halali et al., 2020), poorer mental health, and depression (Richard et al., 2016; Sharpe et al., 2018). As for eating behavior dimensions, dissatisfaction with weight/shape has been associated with symptoms of eating disorders (Askew et al., 2020). Greater weight dissatisfaction has been associated with greater disinhibition of eating (Bond et al., 2001), more emotional eating (Olson, 2017), more dietary restraint (Bond et al., 2001; Heywood and McCabe, 2006; Lattimore, 2005), and greater susceptibility to hunger (Bond et al., 2001). A survey among adults 18–30 years in eight developed countries reported a negative association between levels of emotional eating and restrained eating with body satisfaction, whereas intuitive eating (i.e. eating in response to internal cues of hunger and satiety) was positively associated with body satisfaction (Markey et al., 2023; Tylka and Kroon Van Diest, 2013). In a study assessing dietary restraint by asking participants whether they ate exactly what they wanted or, alternatively, more or less than desired, weight satisfaction was found to be associated with less restrained eating (Kuk et al., 2009).

Evidence for the associations of weight satisfaction and dietary intake in adults is scarce. Middle-aged and older women who were dissatisfied with their weight reported lower intakes of carbohydrates and dietary fiber, regardless of BMI (Chatelan and Carrard, 2021). Compared to women within healthy BMI category who did not wish to lose weight, those who desired to weigh less reported lower energy intake per kg of body weight, lower intake of protein per kg of weight, lower percentage of carbohydrate intake, and higher percentage of lipids intake (Carrard et al., 2018). Other studies have reported either negative or no associations between body weight/body image dissatisfaction and diet quality or food consumption (Carrard et al., 2024; Jackson et al., 2024; Oliveira et al., 2020).

Eating behavior and dietary correlates of weight satisfaction in adulthood has remained relatively understudied, as research on weight/body satisfaction has mainly focused on adolescents and young adults (Christofaro et al., 2015; Duarte et al., 2021; Fredrickson et al., 2015; Markey et al., 2023; Walsh et al., 2009; Xu et al., 2018; Yang et al., 2022). The present study examines whether weight satisfaction in adults with prior weight management experience is associated with eating behavior, weight history, psychological distress, and dietary intake.

## Methods

### Participants

**Figure 1.**
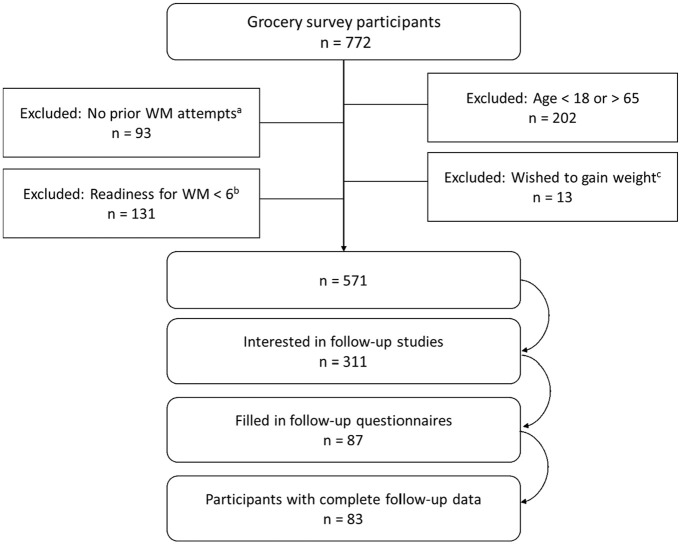
Flow diagram of study participants. (a) Have you tried to lose weight during your lifetime (no/no, but I have been trying to keep my weight stable/yes, 1–2 times/yes, ≥3 times / yes, continuously). (b) How ready are you to make weight management efforts (1, the least ready-10, the readiest). (c) Are you satisfied with your current weight (yes/no, I wish to lose weight/no, I wish to gain weight). WM = weight management.

Participants of the present study are a subgroup of a larger data set in which individuals (*n* = 772) had responded to a grocery survey questionnaire in Jyväskylä and Kuopio, Finland (Halali et al., 2018). Out of individuals who met the study criteria (*n* = 571), 311 declared their interest in taking part in follow-up studies. The present study reports data from individuals who participated in the behavioral analysis study in Jyväskylä (target sample size = 50) and the food choice behavior study in Kuopio (target sample size = 36), and returned fully completed questionnaires (*n* = 83, Figure 1).

Participants (57% women) had a mean (SD) age of 45 (12) years (range 23–65 years), and a mean (SD) BMI of 26.5 (4.5) kg/m^2^ (range 18.4–44.3 kg/m^2^). Participants received the questionnaires by mail and were asked to fill them in and send them back in a prepaid envelope to the researchers within 3 weeks. The study received approval from the Research Ethics Committee of the Northern Savo Hospital District (No. 114/2009). All participants provided informed consent to participate in the study.

### Measures

#### Weight-related measures

In addition to information on age and sex, participants were asked whether they were satisfied with their current weight (yes/no, I wish to lose weight/no, I wish to gain weight), had tried to lose weight during their lifetime (no/no, but I have been trying to keep my weight stable/yes, 1–2 times/yes, ≥3 times/yes, continuously), and how ready they were to make weight management efforts (1, the least ready-10, the most ready). Participants who answered “no, I wish to gain weight” to the weight satisfaction question were excluded (Figure 1), those who responded “yes” were classified as weight-satisfied, and those responding “no, I wish to lose weight” were classified as weight-dissatisfied. Furthermore, participants provided information about their height, weight, current intentions to lose weight (yes/no), frequency of weight loss attempts during their lifetime (none/1–2 times/3–4 times/5–6 times/more than 6 times) and the amount of weight lost in each attempt (less than 5 kg/5-9.9 kg/10–14.9 kg/15–19.9 kg/more than 20 kg) as well as weight changes during the past 12 months (no changes/increased weight/decreased weight).

#### Eating behavior

For assessing eating behavior dimensions, we used the 65-item Three-Factor Eating Questionnaire (TFEQ). The original 51-item TFEQ (Stunkard and Messick, 1985) measures three dimensions of eating behavior: cognitive restraint of eating (21 items; score range 0–21), eating disinhibition (16 items; score range 0–16) and susceptibility to hunger (14 items; score range 0–14). Later, it was modified by adding 14 items to better capture the two distinct subscales of eating restraint: flexible restraint (12 items, defined as a graduated approach to eating and weight control) and rigid restraint (16 items, an all-or-nothing approach to eating; Westenhoefer et al., 1999). Responses to all TFEQ items are recorded as 0 and 1. Items 10, 16, 21, 25, 30, 31, 47, 52, and 58 were reverse-coded. The total score for each scale or subscale is calculated by summing the scores of its individual items. The higher the score, the higher level of that dimension.

When evaluating internal consistency, Cronbach’s alpha for total restraint was 0.77, while the flexible and rigid restraint subscales had Cronbach’s alphas of 0.75 and 0.66, respectively. For total disinhibition, Cronbach’s alpha was 0.80, and for total hunger, it was 0.85. We also analyzed the subscales for disinhibition—habitual susceptibility, emotional susceptibility, and situational susceptibility—and for hunger, which included internal hunger and external hunger (Bond et al., 2001). The Cronbach’s alphas for disinhibition subscales were 0.74, 0.82 and 0.59, respectively. Item 16 (*it is not difficult for me to leave something on my plate*) was poorly correlated with the other items and was therefore excluded from the situational susceptibility and the total disinhibition. The Cronbach’s alphas for internal hunger and external hunger were 0.80 and 0.69, respectively. Item 47 (*how often do you skip dessert because you are not hungry anymore?*) was excluded from the external hunger and the total hunger due to small correlations with the other items. Supplemental Table 1 presents the inter-item correlation matrix for the “situational susceptibility” and the “external hunger” subscales, from which one item was removed from each due to low correlations with other items. Some researchers recommend reporting the ordinal alpha alongside the traditional Cronbach’s alpha for assessing inter-item reliability in categorical response data, as the latter has been argued to underestimate internal consistency for this type of data (Park, 2024). The ordinal alpha values for all scales and subscales of the TFEQ are presented in Supplemental Table 2.

#### Psychological distress

To measure psychological distress, the 12-item General Health Questionnaire, GHQ-12, was used (Goldberg, 1972). The GHQ-12 has shown to be a valid screening tool for common mental health problems in the Finnish population (Holi et al., 2003). The timeframe of this questionnaire concerns the past few weeks, and respondents were asked questions such as “*Have you experienced loss of sleep due to your worries?*.” The Likert scoring system (0–1–2–3) was used (total score range 0–36) as a higher score indicates a greater level of psychological distress. Cronbach’s alpha for the GHQ-12 was 0.85. The ordinal alpha value is presented in Supplemental Table 2.

#### Dietary intake

The semi-quantitative food frequency questionnaire (FFQ) used in this study consists of questions about frequency of use of 155 common Finnish food items during the past 12 months, defined in portion sizes (Paalanen et al., 2006). As an example, it asks participants to indicate frequency of use of one slice of rye bread or a glass of milk with 9 answer options ranging “not at all” to “6 times or more per day.” Since the study population was experienced in weight management, four food groups considered important for weight management were formed: (i) high-fiber cereals (3 items; rye bread, rye crisps, muesli); (ii) low-fiber cereals (2 items; white wheat bread, home-made rolls); (iii) sweets and pastries (11 items, e.g. donut, cake and pastry, cookie and biscuit, ice cream, sugar-sweetened beverages); (iv) fruits and vegetables (27 items, e.g. apple, orange juice, tomato, cauliflower). The average daily intake of these food groups was calculated. Additionally, participants were asked to keep a 4-day dietary record on 3 weekdays and one weekend day. The portion sizes were estimated using household measures. Nutrient intake was analyzed using Diet-32 analysis program (Aivo Finland Oy) and the Finnish food composition database Fineli (National Institute for Health and Welfare). The number of participants included in the dietary analyses was 79, as four individuals did not provide any dietary data.

### Statistical analysis

As the continuous variables did not have normal distributions, that is, showed a significant test of normality (Shapiro–Wilk test, *p* < 0.05), the nonparametric Mann-Whitney test was used for assessing their relationships with weight satisfaction. To measure the associations of categorical variables with weight satisfaction the Chi-squared test was used. Following the Chi-squared test, a post hoc *z*-test with Bonferroni correction was performed to determine which groups differed significantly from each other. To quantify the mean differences in outcome measures between the weight-dissatisfied and weight-satisfied groups, effect sizes were calculated (Cohen, 1988).

Binary logistic regression was utilized to assess the likelihood of weight dissatisfaction in relation to cognitive restraint of eating, eating disinhibition, susceptibility to hunger, and psychological distress. The baseline model was adjusted for age, sex, BMI status, lifetime number of attempts to lose weight, and total energy intake. The Hosmer and Lemeshow goodness of fit index indicated that both the baseline and adjusted regression models were fit enough (*p* = 0.17 and *p* = 0.42, respectively). The subcomponents for cognitive restraint of eating, eating disinhibition, and susceptibility to hunger were not separately included in the model due to multicollinearity. SPSS, version 27 (IBM SPSS Statistics for Windows, Armonk, NY) was used for statistical analyses. *p* < 0.05 was considered statistically significant. Ordinal alpha calculations were performed in R Statistical Software (v 4.4.1; R Core Team, 2024), using packages psych (Revelle, 2024) and polycor (Fox and Dusa, 2022).

## Results

About two-thirds of the participants [*n* = 55/83, 66%, mean (SD) age 44 (12) years] were dissatisfied with their weight and formed the weight-dissatisfied group. The weight-satisfied participants had a mean (SD) age of 47 (11) years. There was no statistical difference in the mean age between weight-satisfied and weight-dissatisfied groups (47 vs 44 years, *p* > 0.05). Prevalence of weight dissatisfaction among women and men were 72% and 58%, respectively (*p* > 0.05). In the weight-satisfied group, 46% were women and 54% were men, while the distribution was 62% and 38% in the weight-dissatisfied group (*p* > 0.05).

### Weight satisfaction and weight history

Current BMI was significantly larger in the weight-dissatisfied group (Table 1). A larger proportion of the weight-dissatisfied group reported prior weight loss attempts and yet were aiming to lose weight, while a much larger proportion of the weight-satisfied group reported having tried to maintain their weight. The proportion of individuals with more recent fluctuation in body weight (increase or decrease) was larger in the weight-dissatisfied group. The weight-dissatisfied group had lost totally more weight over their lifetime than the weight-satisfied group (Table 1).

**Table 1:** Body weight and weight history in the weight-satisfied and weight-dissatisfied groups. Values are reported as mean (SD: standard deviation) and Median (IQR: Interquartile range) for continuous variables and frequency (percentage) for categorical variables.

Variable	Satisfied (*n* = 28)	Dissatisfied (*n* = 55)	*p* *
BMI^1^ (kg/m^2^), Mean (SD)	23.5 (2.9)	28.1 (4.4)	
Median (IQR)	24 (4)	27.1 (5.5)	<**0.001**
BMI category^2^ (*n*, %)			
Normal weight	19 (68)^a^	14 (25)^a^	
Overweight	8 (28)^b^	25 (45)^b^	
Obesity	1 (3)^b^	16 (29)^b^	<**0.001**
Readiness for weight management efforts^3^, Mean (SD)	8.2 (1.2)	7.8 (1.0)	
Median (IQR)	8 (1)	8 (1)	0.054
Number of Lifetime attempts to lose weight (*n*, %)			
None, but trying to keep weight stable	15 (53)^a^	6 (11)^a^	
1–2 times	7 (25)^b^	17 (31)^b^	
≥3 times	4 (14)^b^	15 (27)^b^	
Continuously	2 (7)^b^	17 (31)^b^	<**0.001**
Currently aiming to lose weight (*n*, %)			
Yes	2 (7)^a^	41 (74)^a^	
No	26 (93)^b^	14 (26)^b^	<**0.001**
Changes in weight during the past 12 months (*n*, %)			
No change	11 (39)^a^	5 (9)^a^	
Increased	8 (28)^b^	29 (53)^b^	
Decreased	9 (32)^b^	21 (38)^b^	**0.003**
Lifetime amount of lost weight (kg), Mean (SD)	17.9 (22.1)	39.6 (56.8)	
Median (IQR)	9.9 (26.6)	22.2 (27.3)	**0.009**

* Mann-Whitney test for continuous variables and Chi-squared test for categorical variables.The *p*-values in bold indicate significant differences between the two groups.

^1^Body Mass Index; ^2^Normal weight: 18.5 ≤ BMI ≤ 25 kg/m^2^, overweight: 25 < BMI ≤ 29.9 kg/m^2^, obesity: BMI ≥ 30 kg/m^2^. ^3^Measured on a scale of 1–10 (least ready-most ready), eligible participants to be included in this study were to have a readiness of 6 or more.

Superscript letters indicate whether categories differ significantly from each other based on post hoc *z*-tests. The difference is significant if the letters are different. For example, in both groups of weight-satisfied and weight-dissatisfied, the proportion of normal-weight individuals is significantly different (higher and lower, respectively) from that of individuals with overweight and obesity.

### Weight satisfaction, eating behavior, and psychological distress

Individuals who were dissatisfied with their weight reported more rigid restraint, more eating disinhibition, and higher levels of all the subcomponents of eating disinhibition (habitual, emotional, and situational susceptibility) than the weight-satisfied group (Table 2). Effect sizes indicated large mean differences for rigid restraint (*d* = 0.85), total eating disinhibition (*d* = 1.03), and habitual susceptibility (*d* = 0.87). Mean differences for emotional and situational susceptibility were of medium magnitude (*d* = 0.70 and *d* = 0.63, respectively; Cohen, 1988). The weight-dissatisfied and weight-satisfied groups did not differ in total or flexible restraint of eating, susceptibility to hunger, or psychological distress (*p* > 0.05).

**Table 2.** Cognitive restraint of eating, disinhibition of eating, susceptibility to hunger, and psychological distress in the weight-satisfied and weight-dissatisfied groups.

Variable	Satisfied		Dissatisfied		p*
	(*n* = 28)		(*n* = 55)		
	Mean (SD)	Median (IQR)	Mean (SD)	Median (IQR)	
Cognitive restraint of eating^a^	9.2 (4.1)	9.5 (7.0)	10.4 (4.0)	11.0 (6.0)	0.19
Flexible restraint^b^	5.9 (2.9)	6.0 (5.0)	6.1 (2.7)	6.0 (4.0)	0.77
Rigid restraint^c^	4.5 (2.8)	4.0 (3.0)	6.8 (2.7)	7.0 (4.0)	<**0.001**
Disinhibition^d^	3.7 (2.3)	4.0 (3.7)	7.2 (3.2)	7.0 (6.0)	<**0.001**
Habitual susceptibility^e^	0.6 (1.0)	0 (1.0)	1.8 (1.5)	1.0 (2.0)	<**0.001**
Emotional susceptibility^f^	0.4 (0.9)	0 (0.7)	1.2 (1.2)	1.0 (3.0)	**0.002**
Situational susceptibility^g^	1.3 (1.1)	1.0 (2.0)	2.1 (1.2)	2.0 (2.0)	**0.005**
Hunger^h^	3.5 (2.8)	2.5 (4.0)	4.8 (3.6)	4.0 (5.0)	0.11
Internal hunger^i^	1.2 (1.6)	0.5 (2.0)	1.9 (2.0)	1.0 (3.0)	0.1
External hunger^j^	0.8 (1.1)	1.0 (1.0)	1.6 (1.6)	1.0 (3.0)	0.09
Psychological distress^k^	10.0 (4.1)	10.0 (5.7)	10.8 (4.5)	10.0 (6.0)	0.47

* Mann-Whitney test.

SD: standard deviation; IQR: interquartile range.The *p*-values in bold indicate significant differences between the two groups.

Possible score range: ^a^0–21; ^b^0–12; ^c^0–16; ^d^0–16; ^e^0–5; ^f^0–3; ^g^0–5; ^h^0–14; ^i^0–6; ^j^0–6; ^k^0–36.

Scales “d” and “g” do not include item 16 (*it is not difficult for me to leave something on my plate*), and scales “h” and “j” do not include item 47 (*how often do you skip eating dessert because you are not hungry anymore?*).

a–j Three-Factor Eating Questionnaire (TFEQ-65); ^k^General Health Questionnaire (GHQ-12).

Disinhibition of eating predicted weight dissatisfaction as each unit increase in the level of disinhibition increased the likelihood of weight dissatisfaction by 1.6-folds (Table 3). This finding was independent of age, sex, BMI status, number of weight loss attempts during lifetime, and energy intake. The other eating behavior dimensions or psychological distress did not predict weight dissatisfaction.

**Table 3.** Predictors of weight dissatisfaction among the study participants.

Variable	Unadjusted model OR^a^ (95% CI^b^)	Adjusted model^c^ OR (95% CI)
Cognitive restraint of eating	1.09 (0.95–1.25)	1.09 (0.92–1.30)
Disinhibition of eating	1.73 (1.28–2.33)	**1.64 (1.11–2.41)**
Hunger	0.86 (0.68–1.07)	0.95 (0.71–1.26)
Psychological distress	0.99 (0.87–1.14)	0.97 (0.83–1.14)

a Odds ratio; ^b^Confidence interval; ^c^Regression model is adjusted for age, sex, body mass index (BMI) status, lifetime number of weight loss attempts, and total energy intake.The OR (95% CI) in bold indicates that disinhibition of eating predicts weight dissatisfaction.

Female sex, normal-weight BMI status, and trying to maintain weight during lifetime were selected as the reference categories for sex, BMI status, and lifetime number of attempts to lose weight, respectively.

### Weight satisfaction and dietary intake

The total energy intake for the weight-dissatisfied and the weight-satisfied groups were not significantly different, 1916 (487) kcal and 2083 (707) kcal, respectively (*p* > 0.05). Although fiber intake was significantly lower in the weight-dissatisfied group [21 (7) g vs 27 (12) g, *p* = 0.03], the difference did not remain significant when adjusting for energy intake. The groups did not differ in the intake of macronutrients and sugars, or food groups.

## Discussion

The present study investigated the associations between weight satisfaction and eating behavior, psychological distress, body weight, and dietary intake. Disinhibition of eating predicted weight dissatisfaction. Disinhibition of eating is a broad term that incorporates several types of losing control over eating in response to external and internal stimuli (Bond et al., 2001; Stunkard and Messick, 1985). This study assessed three components of disinhibition—habitual, emotional, and situational susceptibility—and found that all three had higher levels in the weight-dissatisfied group. Habitual susceptibility to disinhibition, which refers to daily circumstances that prone individuals to disinhibited eating, showed a stronger association with weight satisfaction compared to disinhibition due to emotional states (emotional susceptibility) or environmental situations (situational susceptibility; Bond et al., 2001). Disinhibition and habitual susceptibility to disinhibition have been reported to mediate the association between genetic risk of obesity and BMI (Jacob et al., 2018). It seems that more attention should be directed toward addressing disinhibited eating, particularly in the context of general daily life circumstances, among individuals with weight management experience.

We found that weight dissatisfaction was additionally associated with greater rigid restraint of eating, more attempts to lose weight, greater amounts of lifetime weight lost, more recent weight changes and yet a higher current BMI. These findings may indicate a greater tendency to yo-yo dieting and weight cycling in the weight-dissatisfied group, which could increase health risks and mortality (Blake et al., 2013; Kodama et al., 2017; Kuk et al., 2009). Previous studies have indicated a positive association between dieting and weight loss attempts, and higher BMIs (Halali et al., 2020; Sainsbury et al., 2019; Sares-Jäske et al., 2019; Siahpush et al., 2015), forming a cycle of dieting, weight gain, and dissatisfaction with weight. Although the cause-effect direction could not be ascertained, our findings suggest that frequent attempts to lose weight could prompt disinhibited eating (Halali et al., 2020; Marchesini et al., 2004), particularly when accompanied by rigid food control strategies (Vlahoyiannis and Nifli, 2020). Frequent dieting attempts and strict attitudes toward eating, to alter either weight or shape, may lead to overconsumption of food in a variety of situations, weight gain over time, and weight dissatisfaction. However, whether baseline levels of weight dissatisfaction moderate the dynamic association between eating behaviors and weight dissatisfaction should be further studied. It also remains unclear whether individuals who are dissatisfied with their weight and exhibit disinhibition of eating have higher BMIs than those who are weight-dissatisfied with no signs of eating disinhibition.

From the clinical point of view, our findings suggest that it may be relevant to identify individuals who are dissatisfied with their current weight and report a history of weight loss attempts, as they could exhibit symptoms of disordered eating behaviors that impede proper weight management. More precisely, health promotion and weight management programs could consider targeting the interrelations of weight dissatisfaction, dieting behavior, and disordered eating symptoms. Body appreciation, which is the most precise measure of positive body image, contributes to less disordered eating, and more adaptive eating behaviors (Linardon, 2021; Linardon et al., 2022; Messer et al., 2022). There is evidence that cognitive-behavioral therapy (CBT), and acceptance and commitment therapy (ACT) interventions could improve body image and disordered eating outcomes including uncontrolled eating and emotional eating among those with high levels of body dissatisfaction (McLean et al., 2011; Pearson et al., 2012). Moreover, a meta-analysis of prevention programs supported reduced risk of eating disorders mainly through improving body dissatisfaction and influencing dieting behavior (Harrer et al., 2019). These findings may be promising for designing interventions to improve weight dissatisfaction, as weight dissatisfaction could be a reflective measure of body dissatisfaction (Coker and Abraham, 2014). In addition, weight-inclusive approaches that prioritize health and wellbeing over body weight could decrease weight stigma, body dissatisfaction, drive for thinness, and disordered eating behaviors such as eating disinhibition (Bacon et al., 2005; Dugmore et al., 2020).

Psychological distress was not associated with weight satisfaction in the present study. Levels of psychological distress were relatively low, with no differences between weight-dissatisfied and weight-satisfied groups. Previous research has reported associations between poorer mental health, depression and body dissatisfaction (Richard et al., 2016; Silva et al., 2019). Body appreciation has been associated with higher psychological wellbeing and life satisfaction (Swami et al., 2023). The discrepancies in the findings may be partly attributed to differences in study populations and/or use of different tools to measure psychological distress. However, it is utmost important to study psychological distress and psychological wellbeing in the context of weight/body dissatisfaction. This is especially necessary to be addressed in people living with overweight and obesity who are more likely to be dissatisfied with their weight, deal with psychological consequences of obesity, and experience weight bias in society, media, and health systems (Fulton and Srinivasan, 2023; Macho et al., 2023). In clinical settings, addressing psychological wellbeing may improve body image outcomes, disordered eating behaviors, weight control, adopting health-promoting behaviors, and quality of life (Merino et al., 2024; Sairanen et al., 2014).

Regarding dietary intake, weight satisfaction was not associated with total energy intake. The small difference in dietary fiber intake was no longer significant after adjusting for energy intake. Nevertheless, underreporting dietary intake in the weight-dissatisfied group could not be ruled out (Tyrovolas et al., 2016). Moreover, participants of the present study were experienced in weight management and could have been more knowledgeable about the role of diet in health and weight management. Larger studies should research the links between different aspects of diet, diet quality, dietary patterns, and weight satisfaction.

### Strengths and limitations

To our knowledge, the present study is the first among adults in Finland investigating weight satisfaction and its relation to eating behavior dimensions, psychological well-being, weight history, and dietary intake. The extended version of the TFEQ was used to capture the specific components of cognitive restraint of eating, eating disinhibition, and susceptibility to hunger. Although we acknowledge that removing two items from the TFEQ might limit the comparability of our findings to other studies using the original questionnaire, we believe this modification strengthens the robustness of our findings. Furthermore, future studies with larger sample sizes should evaluate the construct validity of the TFEQ-51, as the small sample size and high model complexity in the present study precluded such an analysis.

Data for the present study was derived from community-dwelling adults, who had previous experience in weight management and reported a high level of readiness to make weight management efforts. On the one hand, this criterion could provide real-life perspectives from people with actual weight management experiences and intentions. On the other hand, it could limit the generalizability of the findings as it excluded individuals who had not practiced any conscious weight management efforts, and neither were ready to make efforts in the future yet were dissatisfied with their weight. Small sample size was another important limitation, reducing generalizability of the findings.

Dietary intake was assessed using dietary records and an FFQ, both of which are validated and reliable methods for measuring dietary intake. However, the likelihood of underreporting dietary intake should be acknowledged, as it may arise from underlying sources of bias. Recall bias is a notable concern when using the FFQ, as participants may struggle to accurately recall their intake over an extended period. Additionally, participants may provide socially desirable responses for both the FFQ and dietary record, underreporting unhealthy food consumption or overreporting healthier choices (Gemming and Ni Mhurchu, 2016; Naska et al., 2017; Shim et al., 2014). Tendency to give socially desirable responses applies to other self-report questionnaires as well (Kuncel and Tellegen, 2009). With a Likert scoring model, some respondents may tend to select the extreme response categories while ignoring the middle options, for example, responding “never” or “always” and ignoring other options such as “once a week” (Shou et al., 2022). For the items requiring reverse coding, careless responding may provide answers that are not sensitive to content (Weijters et al., 2013).

The potential presence of eating disorders, such as binge eating or bulimia nervosa, among some participants may have influenced the sample characteristics as these participants may have higher likelihoods of more frequent weight loss attempts and being dissatisfied with their weight. Having an eating disorder may influence how individuals perceive and respond to questions about eating behaviors, by underreporting or overreporting of certain behaviors (Howard et al., 2020; Schoen et al., 2012). Future studies may benefit from screening for eating disorders. Body weight was self-reported; however, self-reported weight has been shown to be a valid measure for assessing weight status at the population level (Moreira et al., 2018). On the other hand, body weight is also prone to underreporting, particularly among individuals with excess weight (Maukonen et al., 2018).

### Conclusions

Dissatisfaction with body weight was associated with more unfavorable dimensions of eating behavior, in particular greater eating disinhibition and rigid restraint of eating. The weight-dissatisfied group had a higher BMI, more prior attempts to lose weight, and more recent weight fluctuations, whereas dietary factors did not differentiate the weight-dissatisfied and weight-satisfied groups. Future studies should incorporate larger sample sizes and explore the directionality of associations between changes in eating behavior dimensions and shifts in weight satisfaction.

## Supplemental Material

sj-docx-1-hpq-10.1177_13591053251338340 – Supplemental material for Weight dissatisfaction is linked to higher eating disinhibition and greater weight fluctuations in adults with weight management experience Supplemental material, sj-docx-1-hpq-10.1177_13591053251338340 for Weight dissatisfaction is linked to higher eating disinhibition and greater weight fluctuations in adults with weight management experience by Faranak Halali, Anja Lapveteläinen, Liisa I Tammela, Sari Hantunen, Raimo Lappalainen, Teuvo Kantanen and Leila Karhunen in Journal of Health Psychology

sj-docx-2-hpq-10.1177_13591053251338340 – Supplemental material for Weight dissatisfaction is linked to higher eating disinhibition and greater weight fluctuations in adults with weight management experience Supplemental material, sj-docx-2-hpq-10.1177_13591053251338340 for Weight dissatisfaction is linked to higher eating disinhibition and greater weight fluctuations in adults with weight management experience by Faranak Halali, Anja Lapveteläinen, Liisa I Tammela, Sari Hantunen, Raimo Lappalainen, Teuvo Kantanen and Leila Karhunen in Journal of Health Psychology
