# Supplementary material for: Weight dissatisfaction is linked to higher eating disinhibition and greater weight fluctuations in adults with weight management experience
Source: J Health Psychol. 2025 May 29;31(4):1425–38. doi: 10.1177/13591053251338340 (PMC12960739; doi:10.1177/13591053251338340)
Supplement: sj-docx-1-hpq-10.1177_13591053251338340 – Supplemental material for Weight dissatisfaction is linked to higher eating disinhibition and greater weight fluctuations in adults with weight management experience [file sj-docx-1-hpq-10.1177_13591053251338340.docx]

**Supplementary Table 1.1** Correlation matrix^*^ for the ‘situational susceptibility’ items

|  | Item 2 | Item 7 | Item 13 | Item 15 | Item 16 |
| --- | --- | --- | --- | --- | --- |
| Item 2 | 1 | .258 | .334 | .151 | .003 |
| Item 7 | .258 | 1 | .291 | .295 | -.305 |
| Item 13 | .334 | .291 | 1 | .381 | -.188 |
| Item 15 | .151 | .295 | .381 | 1 | -.076 |
| Item 16 | .003 | -.305 | -.188 | -.076 | 1 |

^*^Based on Pearson correlation

**Supplementary Table 1.2** Correlation matrix^*^ for the ‘external hunger’ items

|  | Item 8 | Item 19 | Item 22 | Item 26 | Item 41 | Item 47 |
| --- | --- | --- | --- | --- | --- | --- |
| Item 8 | 1 | 0.278 | 0.386 | 0.364 | -0.331 | -0.005 |
| Item 19 | 0.278 | 1 | 0.460 | 0.253 | -0.397 | 0.250 |
| Item 22 | 0.386 | 0.460 | 1 | 0.364 | -0.331 | 0.319 |
| Item 26 | 0.364 | 0.253 | 0.364 | 1 | -0.391 | 0.162 |
| Item 41 | -0.331 | -0.397 | -0.331 | -0.391 | 1 | -0.151 |
| Item 47 | -0.005 | 0.250 | 0.319 | 0.162 | -0.151 | 1 |

*Based on Pearson correlation
