# Supplementary material for: Weight dissatisfaction is linked to higher eating disinhibition and greater weight fluctuations in adults with weight management experience
Source: J Health Psychol. 2025 May 29;31(4):1425–38. doi: 10.1177/13591053251338340 (PMC12960739; doi:10.1177/13591053251338340)
Supplement: sj-docx-2-hpq-10.1177_13591053251338340 – Supplemental material for Weight dissatisfaction is linked to higher eating disinhibition and greater weight fluctuations in adults with weight management experience [file sj-docx-2-hpq-10.1177_13591053251338340.docx]

**Supplementary Table 2** Cronbach’s alpha and ordinal alpha values of the measured outcomes.

|  | Cronbach’s alpha^1^ | Ordinal alpha^2^ |
| --- | --- | --- |
|  |  |  |
| Cognitive restraint of eating^a^ | 0.77 | 0.85 |
| Flexible restraint^b^ | 0.75 | 0.83 |
| Rigid restraint^c^ | 0.66 | 0.80 |
| Disinhibition^d^ | 0.80 | 0.88 |
| Habitual susceptibility^e^ | 0.74 | 0.84 |
| Emotional susceptibility^f^ | 0.82 | 0.94 |
| Situational susceptibility^g^ | 0.59 | 0.80 |
| Hunger^h^ | 0.85 | 0.92 |
| Internal hunger^i^ | 0.80 | 0.91 |
| External hunger^j^ | 0.69 | 0.86 |
| Psychological distress^k^ | 0.85 | 0.90 |

^1^Based on Pearson correlation; ^2^Based on tetrachoric correlation for items a-j, and polychoric correlation for item k.

Possible score range: ^a^0-21; ^b^0-12; ^c^0-16; ^d^0-16; ^e^0-5; ^f^0-3; ^g^0-5; ^h^0-14; ^i^0-6; ^j^0-6; ^k^0-36.

Scales ‘d’ and ‘g’ do not include item 16 (*it is not difficult for me to leave something on my plate*), and scales ‘h’ and ‘j’ do not include item 47 (*how often do you skip eating dessert because you are not hungry anymore?*).

^a-j^Three-Factor Eating Questionnaire (TFEQ-65); ^k^General Health Questionnaire (GHQ-12).
